# Inhibition of ACSL4 Attenuates Behavioral Deficits by Regulating Ferroptosis in a Murine Model of Systemic Lupus Erythematosus

**DOI:** 10.3390/ijms26083553

**Published:** 2025-04-10

**Authors:** Mengdi Jiang, Heng Cao, Weiqian Chen, Ye Yu, Jin Lin

**Affiliations:** Department of Rheumatology, The First Affiliated Hospital of Zhejiang University School of Medicine, Hangzhou 310003, China

**Keywords:** systemic lupus erythematosus, neuropsychiatric syndrome, ACSL4, GPX4, ferroptosis

## Abstract

Neuropsychiatric systemic lupus erythematosus (NPSLE) is a disorder with a poor prognosis characterized by psychiatric and neurological manifestations directly associated with systemic lupus erythematosus (SLE). Neutrophil ferroptosis has been identified as a significant contributor to neutropenia and disease progression in SLE, but its role in NPSLE remains unclear. Female MRL/lpr and MRL/Mpj mice were used. The selective ferroptosis inhibitor liproxstatin-1 and the acyl-CoA synthetase long-chain family member 4 (ACSL4) inhibitor rosiglitazone were administered separately. Assessments included behavioral testing, transmission electron microscopy (TEM), ELISA, Western blotting, RT-PCR, and Nissl staining. Our data showed that neurons in the brain parenchyma undergo ferroptosis, with decreased glutathione peroxidase 4 (GPX4) expression and increased levels of lipid peroxidation indicators and have the typical morphology of ferroptosis confirmed by transmission electron microscopy. Selective ferroptosis inhibitor liproxstatin-1 attenuated the neuropsychiatric manifestations, including depression-like and impulsive behaviors, of MRL/lpr mice. ACSL4 is the main enzyme in lipid metabolism. Our study further found that the utilization of rosiglitazone by inhibiting ACSL4 could also significantly attenuate neuropsychiatric manifestations of MRL/lpr mice. Moreover, blocking ACSL4 might considerably boost GPX4 levels and decrease lipid peroxidation indicators in NPSLE, with reduced neuronal damage, as well as reduced neuroinflammation. This study concluded that inhibiting ACSL4 could facilitate the recuperation of behavioral deficits by suppression of ferroptosis in NPSLE, implying that ACSL4 might be a potential new therapeutic focus for NPSLE.

## 1. Introduction

Systemic lupus erythematosus (SLE) is an autoimmune disorder characterized by dysregulated immune function, leading to the loss of self-antigen tolerance [1]. Patients with SLE have heterogeneous manifestations and multi-organ impairment involving the joints, kidneys, nervous system, and hematopoietic organs [2]. Neuropsychiatric manifestations occur in 15–75% of lupus patients and almost encompass the entire spectrum of psychiatric or neurological dysfunction [3]. Neuropsychiatric systemic lupus erythematosus (NPSLE) syndromes involve focal or diffuse central nervous system (CNS) disorders and peripheral nervous system disorders, with neuropsychiatric manifestations ranging from acute confusional state, psychosis, and seizures to strokes and thrombotic events, accounting for considerable morbidity and mortality in lupus patients [4].

As of now, the pathogenesis of NPSLE is unclear. Though studies have reported that pathogenic antibodies and various cytokines and complement proteins may lead to tissue injury and neuroinflammation [5,6,7], accumulating evidence has indicated that, in lupus, autoantibodies may not be the main mediators and have no direct relationship with brain illness [8,9,10]. Notably, neuropsychiatric manifestations such as emotional abnormalities are usually apparent at the time of the lupus diagnosis in patients and are discovered in mice before gross serological pathology [11,12]. These suggest that, rather than peripheral autoimmunity factors alone, primary abnormal CNS mechanisms are involved in NPSLE pathogenesis. Although NPSLE mechanisms are complex, neuronal death and destruction are key pathogenic factors. A series of consequences caused by neuronal death, such as inflammation and blood–brain barrier disruption, can aggravate the symptoms of NPSLE [13,14].

Ferroptosis, a newly identified pattern of programmed cell death, is defined by iron overload and the formation of lipid-reactive oxygen species (ROS) that lead to necrosome- and caspase-independent cell death [15]. Our recent study has demonstrated that neutrophil ferroptosis plays a key role in lupus. Autoantibodies and cytokines could induce neutrophils to undergo ferroptosis and exacerbate lupus phenotypes [16]. Based on the knowledge that ferroptosis was previously reported in neurodegenerative disorders such as Alzheimer’s disease, Parkinson’s disease, and hemorrhagic stroke [17,18,19], we investigated whether ferroptosis has an influence on NPSLE and the underlying mechanism behind it.

Acyl-CoA synthetase long-chain family member 4 (ACSL4) is a key enzyme in lipid metabolism. Studies found that it could convert free arachidonic acid (AA) into arachidonoyl-CoA11 to generate lipid hydroperoxides, a procedure strongly linked to ferroptosis [20,21,22,23]. Furthermore, pharmacological or genetic deactivation of ACSL4 triggers an anti-ferroptotic rescue pathway, suggesting that ACSL4 might be an objective for the inhibition of ferroptosis [21,23]. Even though ACSL4 is recognized as a critical modulator and significant contributor to ferroptosis [24,25], its specific role in NPSLE remains poorly characterized. Therefore, further investigation into the function of ACSL4 in NPSLE and its relationship with ferroptosis is warranted.

This study demonstrated that ferroptosis is a crucial inducer for the development of NPSLE, and inhibition of ferroptosis facilitates neuropsychiatric behavioral improvement in NPSLE. In parallel, we identified, in a model of NPSLE, that ACSL4 modulates ferroptosis efficiently, and the blockage of ACSL4 promotes neuropsychiatric behavioral rehabilitation in an anti-ferroptotic way.

## 2. Results

### 2.1. Ferroptosis Is Prevalent in NPSLE

Autoimmune mouse models, such as MRL/Mpj-Fas*^lpr^* (MRL/*lpr*), spontaneously develop SLE-like syndromes and can be used to investigate the pathogenesis of NPSLE in an animal model [12].

**Figure 1:**
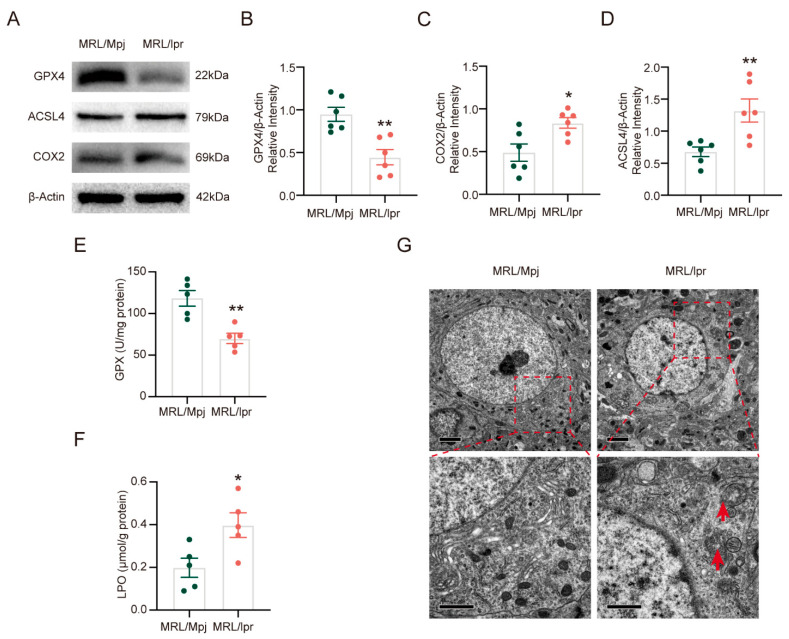
Ferroptosis is prevalent in NPSLE. (**A**) The protein levels of GPX4, COX2, and ACSL4 in MRL/Mpj and MRL/*lpr* mice at 20 weeks, respectively. (**B**–**D**) Quantification of relative expression of GPX4, COX2, and ACSL4; *n* = 6 in each group. (**E**,**F**) Quantification of GPX activity and LPO levels; *n* = 5 in each group. * *p* < 0.05, ** *p* < 0.01 vs. MRL/Mpj. (**G**) Representative transmission electron microscopy images of MRL/Mpj and MRL/*lpr* mice are shown. The red arrows show that the outer mitochondrial membrane of neurons has been damaged and that the number of mitochondrial cristae has diminished or vanished. Upper bar = 2 μm, lower bar = 1 μm, *n* = 3 each group.

To clarify the presence of ferroptosis in NPSLE, certain key ferroptosis-related markers were tested. Glutathione peroxidase 4 (GPX4), a key ferroptosis regulatory protein, was found to be significantly decreased in the brain in lupus (Figure 1A,B). Cyclooxygenase 2 (COX2), which acts as a main enzyme in the preliminary synthesis of prostaglandins from AA, is used as a biomarker of ferroptosis. Compared with the MRL/Mpj mice, the expression of COX2 and ACSL4 in the brain was significantly increased in MRL/*lpr* mice. (Figure 1A,C). Consistent with this, compared with the control group, the expression of ACSL4 was increased in the brain in the MRL/*lpr* group (Figure 1A,D). GPX activity was significantly decreased in the brain of SLE, similar to western blotting findings (Figure 1E), and the level of lipid peroxidation (LPO) was significantly increased in the brain of SLE (Figure 1F). Ferroptosis has a unique morphology in mitochondria [15]. Then, transmission electron microscopy was utilized to investigate the neuronal morphological characteristics. Neurons in the brains of SLE mice exhibited standard morphological features of ferroptosis, including loss of mitochondrial cristae, formation of mitochondrial vacuoles, and damage to the outer mitochondrial membrane (Figure 1G).

All of the above results indicate that ferroptosis is prevalent in NPSLE.

### 2.2. Liproxstatin-1 Attenuates Neuropsychiatric Manifestations of NPSLE in MRL/lpr Mice

**Figure 2:**
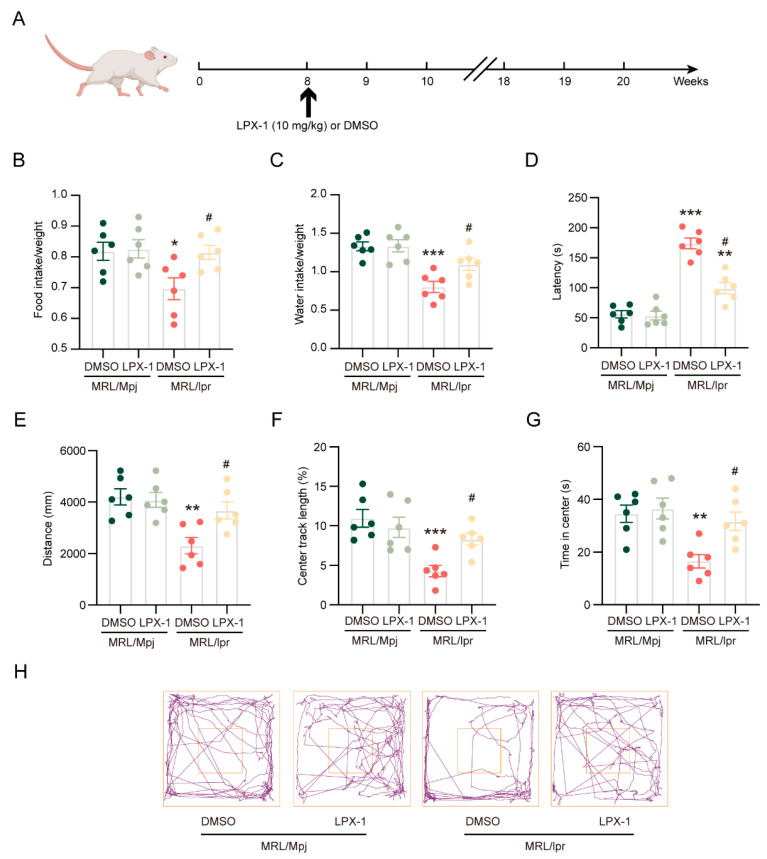
Liproxstatin-1 attenuates the neuropsychiatric manifestations of NPSLE. (**A**) MRL/*lpr* mice were intraperitoneally administered 10 mg/kg liproxstatin-1 every other day for 12 weeks. (**B**,**C**) Quantification of daily food and water intake; *n* = 6 in each group. (**D**) Quantification of the tail suspension test; *n* = 6 in each group. (**E**–**G**) Quantification of total distance, center distance, and time spent in the center area during the open field test; *n* = 6 in each group. (**H**) The representatibe image of the open field test. * *p* < 0.05, ** *p* < 0.01, *** *p* < 0.001 vs. MRL/Mpj + DMSO group; # *p* < 0.05 vs. MRL/*lpr* + DMSO group.

Liproxstatin-1 has been identified as an effective and specific ferroptosis inhibitor [26]. To determine the impact of ferroptosis on neuropsychiatric symptoms in NPSLE, 8-week-old MRL/*lpr* mice were administered liproxstatin-1, and behavioral tests were conducted at 20 weeks of age (Figure 2A). The daily intake of food (Figure 2B) and water (Figure 2C) in MRL/*lpr* mice was significantly lower than that in MRL/Mpj mice, and these behaviors were improved with liproxstatin-1 treatment (Figure 2B,C). The tail suspension test (TST) is used to assess depressive behavior by quantifying the duration that mice remain immobile instead of attempting to break free while suspended by their tails with tape. The duration of immobility was significantly longer in MRL/*lpr* mice compared to the control group. However, immobility was less in liproxstatin-1-treated MRL/*lpr* mice than in vehicle-treated model mice (Figure 2D). The open field test (OFT) is a standard measure of exploratory behavior that assesses some aspects of emotionality, such as anxiety. Based on the OFT, MRL/*lpr* mice explored significantly shorter horizontal distances compared to MRL/Mpj mice in the open field (Figure 2E), with decreased distances (Figure 2F) and time spent (Figure 2G) in the center. The above-mentioned manifestations were markedly improved by liproxstatin-1 treatment (Figure 2E–H).

These findings suggest that liproxstatin-1 has positive impacts on the neuropsychiatric manifestations in MRL/*lpr* mice.

### 2.3. Liproxstatin-1 Inhibits Ferroptosis and Alleviates Lipid Peroxidation in NPSLE

**Figure 3:**
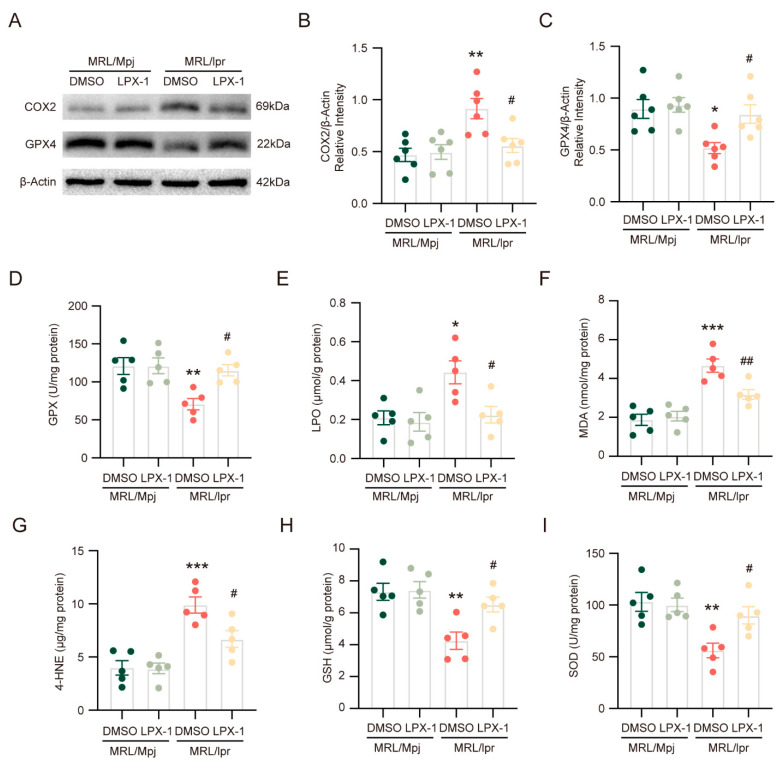
Liproxstatin-1 inhibits ferroptosis to alleviate lipid peroxidation in NPSLE. (**A**) The representative images of the protein levels of COX2 and GPX4. (**B**,**C**) Quantification of COX2 and GPX4 relative expression; *n* = 6 in each group. (**D**–**I**) Quantification of GPX activity, LPO level, MDA level, 4-HNE level, GSH level, and SOD level; *n* = 5 in each group. * *p* < 0.05, ** *p* < 0.01, *** *p* < 0.001 vs. MRL/Mpj + DMSO group; # *p* < 0.05, ## *p* < 0.01 vs. MRL/*lpr* + DMSO group.

Compared with the DMSO treatment, liproxstatin-1 treatment significantly reversed the decreased expression of GPX4 in MRL/*lpr* mice (Figure 3A,C). Similarly, COX2 expression was decreased in MRL/*lpr* mice with liproxstatin-1 treatment compared with DMSO treatment (Figure 3A,B). Consistent with this, GPX activity, GSH level, and superoxide dismutase (SOD) level were significantly higher in the MRL/*lpr* mice treated with liproxstatin-1 (Figure 3D,H,I), while LPO level, malonaldehyde (MDA) level, and 4-HNE level, valuable lipid peroxidation assessment indicators, were significantly lower in the MRL/*lpr* mice treated with liproxstatin-1 (Figure 3E–G).

The above results indicate that liproxstatin-1 alleviates lipid peroxidation and inhibits ferroptosis in NPSLE.

### 2.4. Inhibition of ACSL4 Ameliorates Neuropsychiatric Manifestations of NPSLE in MRL/lpr Mice

**Figure 4:**
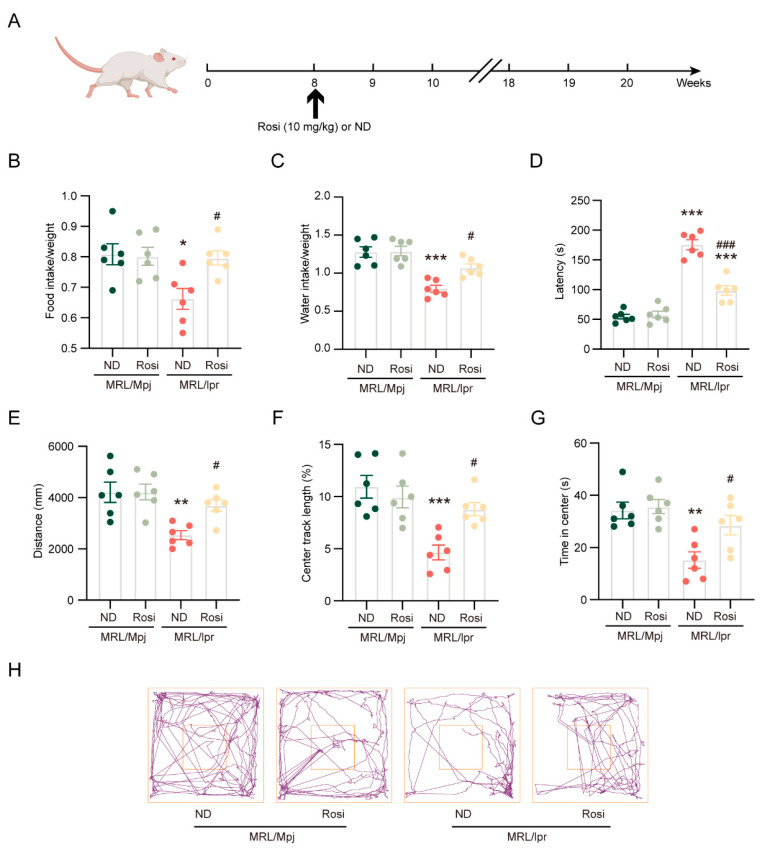
Inhibition of ACSL4 ameliorates neuropsychiatric manifestations of NPSLE (**A**) MRL/*lpr* mice were administered 10 mg/kg/day rosiglitazone with a normal diet for 12 weeks. (**B**,**C**) Quantification of daily food and water intake; *n* = 6 in each group. (**D**) Quantification of the tail suspension test; *n* = 6 in each group. (**E**–**G**) Quantification of total distance, center distance, and time spent in the center area in the open field test; *n* = 6 in each group. (**H**) The representative image of the open field test. * *p* < 0.05, ** *p* < 0.01, *** *p* < 0.001 vs. MRL/Mpj + ND group; # *p* < 0.05, ### *p* < 0.001 vs. MRL/*lpr* + ND group.

Rosiglitazone is identified as an effective ACSL4 inhibitor [23]. To assess the impact of ACSL4 on neuropsychiatric manifestations in NPSLE, MRL/*lpr* mice were given rosiglitazone administration, and behavioral tests were performed at the 20th week (Figure 4A). Daily intake of food (Figure 4B) and water (Figure 4C) was much lower in MRL/*lpr* mice compared to MRL/Mpj mice, and these behaviors were improved with rosiglitazone treatment (Figure 4B,C). The TST revealed that MRL/*lpr* mice were considerably more immobililty than MRL/Mpj mice; the MRL/*lpr* mice treated with rosiglitazone exhibited less immobile than the model mice with a normal diet (Figure 4D). MRL/*lpr* mice traveled shorter horizontal distances than MRL/Mpj mice in the open field test (Figure 4E), with shorter distances (Figure 4F) and less time spent (Figure 4G) in the area. The above abnormal manifestations were significantly ameliorated by rosiglitazone (Figure 4E–H).

These findings suggest that the inhibition of ACSL4 has beneficial effects on neuropsychiatric manifestations in MRL/*lpr* mice.

### 2.5. Inhibition of ACSL4 Suppresses Ferroptosis and Lipid Peroxidation in NPSLE

**Figure 5:**
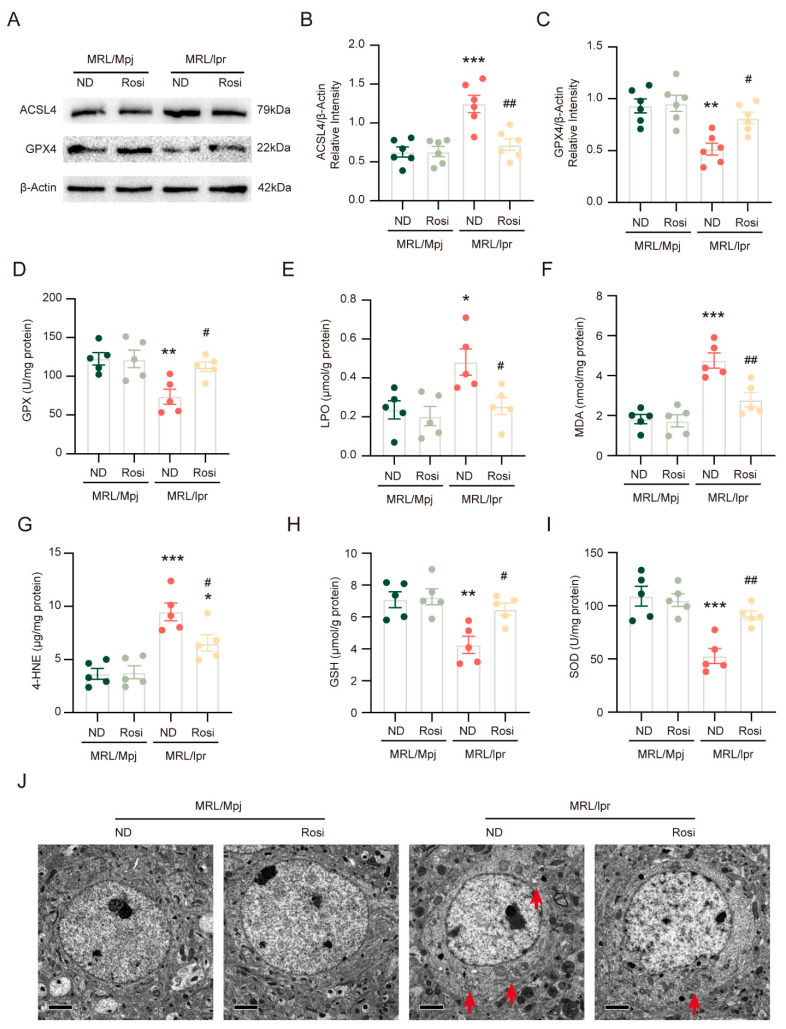
Inhibition of ACSL4 suppresses ferroptosis and lipid peroxidation in NPSLE. (**A**) The representative images of the protein levels of GPX4 and COX2. (**B**,**C**) Quantification of GPX4 and COX2 relative expression; *n* = 6 in each group. (**D**–**I**) Quantification of GPX activity, LPO level, MDA level, 4-HNE level, GSH level, and SOD level; *n* = 5 in each group. * *p* < 0.05, ** *p* < 0.01, *** *p* < 0.001 vs. MRL/Mpj + ND group; # *p* < 0.05, ## *p* < 0.01 vs. MRL/*lpr* + ND group. (**J**) Representative transmission electron microscopy images. The red arrows show that the outer mitochondrial membrane of neurons has been damaged and that the number of mitochondrial cristae has diminished or vanished. Bar = 2 μm.

Compared with the normal diet controls, rosiglitazone treatment significantly reversed the increased expression of ACSL4 in MRL/*lpr* mice (Figure 5A,B). Similarly, the expression of GPX4 in MRL/*lpr* mice with rosiglitazone treatment was increased compared with normal diet treatment (Figure 5A,C). Consistent with this, GPX activity, GSH levels, and SOD levels were significantly higher in the MRL/*lpr* mice treated with rosiglitazone (Figure 5D,H,I), while LPO levels, MDA levels, and 4-HNE levels were significantly decreased in the MRL/*lpr* mice with rosiglitazone treatment (Figure 5E–G). In addition, mitochondrion in the brains of MRL/*lpr* mice with rosiglitazone treatment exhibited decreased morphological features of ferroptosis (Figure 5J).

The above results indicate that rosiglitazone inhibits ferroptosis and lipid peroxidation in NPSLE.

### 2.6. Inhibition of ACSL4 Prevents Neuronal Death and Reverses the Inflammatory Microenvironment in the Brain of MRL/lpr Mice

**Figure 6:**
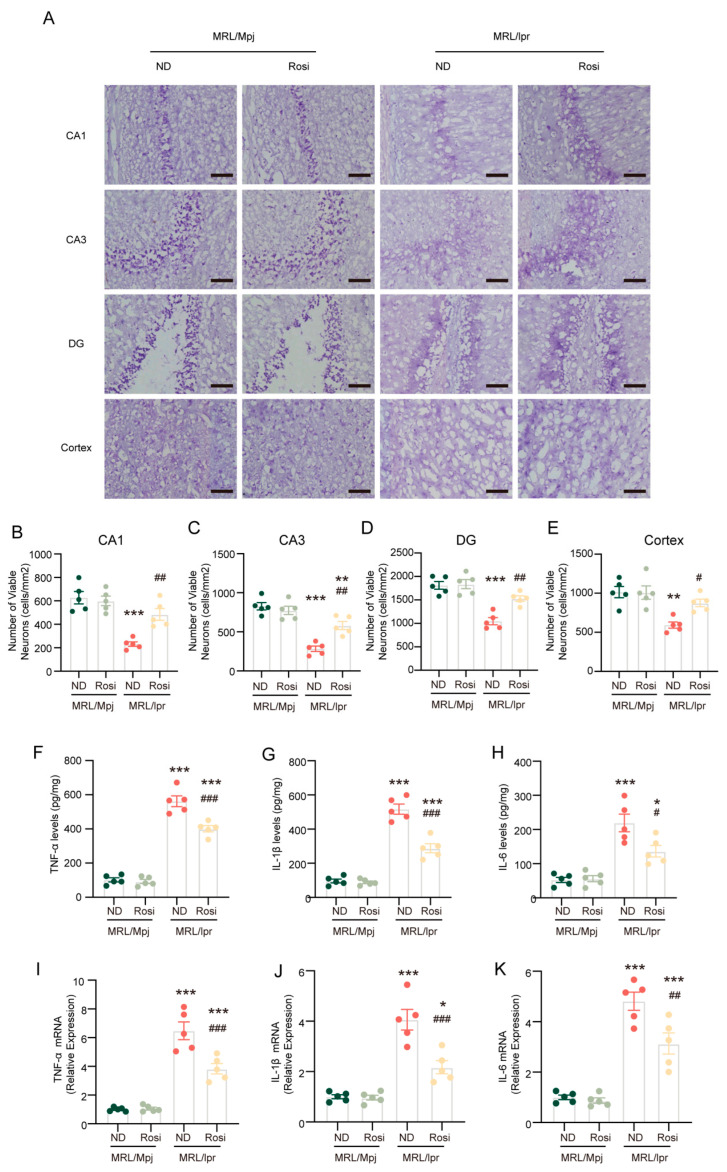
Inhibition of ACSL4 prevents neuronal death and reverses the inflammatory microenvironment in MRL/*lpr* mice. (**A**) The representative images of the Nissl staining in the hippocampus and cortex. Bar = 50 μm. (**B**–**E**) Quantification of surviving neurons in the hippocampus and cortex; *n* = 5 in each group. (**F**–**H**) Quantification of ELISA levels of inflammatory markers; *n* = 5 in each group. (**I**–**K**) Quantification of relative mRNA levels of inflammatory marker genes; *n* = 5 in each group. * *p* < 0.05, ** *p* < 0.01, *** *p* < 0.001 vs. MRL/Mpj + ND group; # *p* < 0.05, ## *p* < 0.01, ### *p* < 0.001 vs. MRL/*lpr* + ND group.

Nissl staining was conducted to evaluate the ability of rosiglitazone to inhibit neuronal degeneration. Mouse brain slices obtained from the MRL/*lpr* mice exhibited significantly fewer surviving neurons in the hippocampal regions (CA1, CA3, and dentate gyrus (DG)) and cortex than MRL/Mpj mouse brain slices, whereas rosiglitazone treatment inverted this reduction (Figure 6A–E). Inflammatory cytokines are crucial to the pathogenesis of NPSLE. Our data found that brain tissues of MRL/*lpr* mice contained higher levels of inflammatory cytokines, such as TNF-α (Figure 6F), IL-1β (Figure 6G), and IL-6 (Figure 6H). Both the protein concentrations (Figure 6F–H) and mRNA levels (Figure 6I–K) of these inflammatory cytokines were significantly reduced following administration of rosiglitazone.

The above results indicate that rosiglitazone inhibits neuronal death and reverses the inflammatory microenvironment in NPSLE.

## 3. Discussion

**Figure 7:**
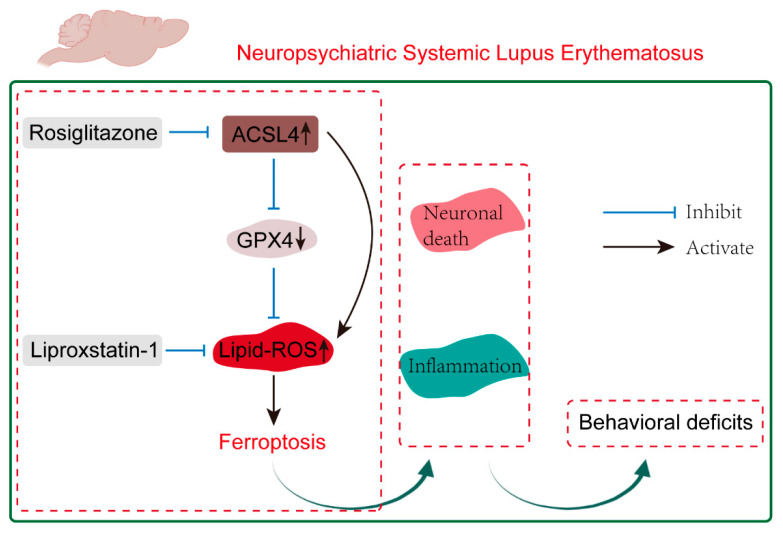
The role of ferroptosis in NPSLE. ACSL4 induces ferroptosis by inhibiting GPX4, which reduces the rise of lipid ROS. By inhibiting ferroptosis, blocking ACSL4 with rosiglitazone greatly accelerates the recovery of behavioral abnormalities in NPSLE.

The MRL/lpr mouse model is a typical and widely studied spontaneous murine model of SLE, characterized by profound autoimmune and lymphoproliferative features due to a homozygous mutation in the Fas gene. In MRL/*lpr* mice, the autoantibody surge phase begins at 8 weeks of age, with immune disorders and a cytokine storm, resulting in a series of cascade reactions. Our results demonstrated the distinct ferroptosis manifestation in the brain parenchyma of MRL/*lpr* mice. The selective ferroptosis inhibitor liproxstatin-1 attenuated neuropsychiatric manifestations, including impulsive and depression-like behaviors of MRL/*lpr* mice, with reduced lipid peroxidation indicators in the brain parenchyma. Furthermore, to explore the underlying mechanisms, we found that the ferroptosis-related protein ACSL4 was significantly increased in MRL/*lpr* mice. The inhibition of ACSL4 by rosiglitazone could significantly attenuate impulsive and depression-like behaviors in MRL/*lpr* mice, with increased expression of GPX4 and decreased lipid peroxidation indicators in the brain parenchyma. Furthermore, rosiglitazone could inhibit neuronal death and reverse the inflammatory microenvironment in NPSLE. Collectively, our results showed that the inhibition of ACSL4 has beneficial effects on the NPSLE model via suppression of ferroptosis, indicating that ACSL4 might be a promising new potential therapeutic for NPSLE (Figure 7).

The CNS is among the major affected organs in patients with SLE, with affected patients having high mortality in the SLE population [27]. In addition to the previous knowledge that neuropsychiatric symptoms are passive injuries due primarily to secondary factors (e.g., brain parenchymal injury or vasculitis) [6], recent studies suggest that neuropsychiatric damage arises earlier, well before the SLE diagnosis compared to other lupus manifestations, and occurs along distinct pathogenic pathways [27]. Furthermore, early recovery from this systemic disease in multiple mouse models of SLE and immunization fails to prevent the occurrence of NPSLE [10]. Consequently, the mechanism underlying the primary NPSLE symptoms is not completely comprehended. In 2022, researchers revealed that, prior to overt peripheral lupus pathology, lupus mice exhibited persistent phagocytic microglial reactivation and significant anxiety-like behavioral patterns, and neuronal restoration of Nr4a1 or antibody inhibition of C1q improved neuropsychiatric manifestations [13].

Ferroptosis is a new modality of cell death induced by lipid peroxidation that varies greatly in morphology, biochemistry, and genetics from apoptosis and autophagy [28]. Recent research indicates that ferroptosis is implicated in the pathophysiology of Alzheimer’s disease, hemorrhagic stroke, and other disorders [29,30,31,32,33]. Our previous study found that liproxstatin-1 treatment in MRL/*lpr* mice significantly mitigated disease progression by inhibition of ferroptosis, reduced the production of autoantibodies, enhanced serum complement component 3, and decreased lymphadenopathy, splenomegaly, and lupus nephritis severity [16]. However, the role of ferroptosis in NPSLE remains poorly understood. Emerging evidence has established that neuropsychiatric manifestations in SLE correlate with neuronal degeneration and cell death [13,14,34]. Our study showed that ferroptosis was prevalent in the brain parenchyma in MRL/*lpr* mice and liproxstatin-1 attenuated impulsive and depression-like behaviors, with reduced lipid peroxidation indicators in the brain parenchyma.

GPX4, currently recognized as a central repressor of ferroptosis [35], is commonly utilized as a molecular biomarker to detect ferroptosis in in vitro or in vivo investigations [36]. Our findings revealed that in NPSLE, the ferroptosis biomarkers COX2 and GPX4 were altered according to the TEM-identified ferroptosis. TEM has been considered a reliable and sensitive approach for discovering ferroptosis visually [37,38]. TEM ultramorphological features of ferroptosis present ruptured and vacuolated cell membranes, increased bilayer density, reduced mitochondrial volume, normal-sized nuclei, reduced or absent mitochondrial cristae, and absence of chromatin cohesion. Moreover, the levels of GSH, GPX activity, SOD, LPO, 4-HNE, and MDA have been identified as crucial indicators in measuring the extent of lipid peroxidation and determining whether ferroptosis exists in NPSLE.

In this study, the underlying mechanism of ferroptosis in NPSLE was investigated. Recent research indicates that the expression of ACSL4 positions it as a promising biomarker to monitor ferroptosis [36]. ACSL4 is responsible for the CoA esterification of free fatty acids, and the synthesis of acyl-CoA prompts lipid peroxidation via the activation of the respective fatty acids [39]. Recent research demonstrates that high ACSL4 expression renders cells more susceptible to ferroptosis by favorably catalyzing polyunsaturated fatty acids, like AA, and influencing the composition of cellular lipids [21]. Moreover, ACSL4-deficient cells are refractory to ferroptosis induced by inactivation of GPX4, which indicates that ACSL4 might be a ferroptosis target [20,21]. To date, the function of ACSL4-mediated ferroptosis in numerous diseases, including tumor growth and intestinal ischemia/reperfusion, has been investigated [21,23]. According to our understanding, this is the first study to report that ACSL4 regulates ferroptosis in NPSLE. In the present study, we evaluated behavioral manifestations in animals and found that inhibition of ACSL4 alleviated psycho-behavioral manifestations. Notably, our findings demonstrated that the use of rosiglitazone by suppressing ACSL4 dramatically ameliorated the brain damage in lupus mice compared to control mice. In conclusion, ACSL4 inhibition enhances the recuperation of behavioral functions in NPSLE, and ACSL4 might be the potential focus of NPSLE treatment. In this experiment, the administration of rosiglitazone significantly lowered the LPO level as well as significant biomarkers of lipid peroxidation, and improved the GPX4 expression, suggesting the improvement of rosiglitazone application in NPSLE is mediated through its inhibitory effect on lipid peroxidation and ferroptosis.

Cytokines, expressed at low levels in the CNS by neurons, astrocytes, oligodendrocytes, and microglia, along with other immune factors, are essential for modulating brain development. Dysregulated production could contribute to multiple cognitive and mood disorders [40]. Accumulating evidence shows that abnormally increased proinflammatory chemokines and cytokines, such as TNF-α, IL-1β, and IL-6, identified in the brain have pathogenic roles in NPSLE patients and trigger tissue injury, which is consistent with our findings [41]. Our data further show that inhibition of ACSL4 in NPSLE significantly reduces neuronal death as well as inhibits neuroinflammation, alleviating the neuropsychiatric manifestations of NPSLE. Uncertainty exists as to whether ACSL can improve behavioral recovery in NPSLE via mechanisms besides the ferroptosis pathway; this is what we shall focus on in our future studies.

## 4. Materials and Methods

### 4.1. Animals

Female MRL/Mpj-Fas*^lpr^* (MRL/*lpr*) and MRL/Mpj mice were purchased from Shanghai Slack Laboratory and kept in pathogen-free conditions with a 12:12-h dark/light cycle and unlimited access to water and food.

### 4.2. Experimental Group Design

#### 4.2.1. Experiment 1

Female MRL/*lpr* and MRL/Mpj mice were assigned to two different groups, the MRL/Mpj group and the MRL/*lpr* group, for enzyme-linked immunosorbent assay (ELISA) (*n* = 5), transmission electron microscopy (TEM) (*n* = 3), and western blotting (*n* = 6).

#### 4.2.2. Experiment 2

Mice were divided into four groups, including the MRL/Mpj + Dimethyl sulfoxide (DMSO) group, the MRL/Mpj + Liproxstatin-1 (LPX-1) group, the MRL/*lpr* + DMSO group, and the MRL/*lpr* + LPX-1 group, for the assessment of behavioral testing (*n* = 6), western blotting (*n* = 6), and ELISA (*n* = 5).

#### 4.2.3. Experiment 3

Mice were divided into four groups, including the MRL/Mpj + normal diet (ND) group, the MRL/Mpj + Rosiglitazone (Rosi) group, the MRL/*lpr* + ND group, and the MRL/*lpr* + Rosi group, for the assessment of behavioral testing (*n* = 6), ELISA (*n* = 5), western blotting (*n* = 6), Nissl staining (*n* = 5), and reverse transcription–polymerase chain reaction (RT-PCR) (*n* = 5).

### 4.3. Drug Administration

In this study, liproxstatin-1 (MedchemExpress) was used as the inhibitor of ferroptosis [16], and rosiglitazone was used as the inhibitor of ACSL4 [42]. Mice were given 10 mg/kg liproxstatin-1 (dissolved in 2% DMSO) or 2% DMSO via intraperitoneal administration on alternate days until they were 20 weeks old, beginning at 8 weeks of age [16]. Mice were provided with either a standard diet or a standard diet supplemented with 10 mg/kg/day rosiglitazone (MedchemExpress) until they were 20 weeks old, beginning at 8 weeks of age [43].

### 4.4. Water and Food Intake

During the 20th week of life, food and water intake were recorded. Over the course of 5 days, the daily food intake was determined by measuring the decrease in food pellets (originally weighing 12–14 g) on the cage floor every 24 h. The daily water intake was recorded as the difference in volume of water-filled leak-proof syringes during each 24-h period.

### 4.5. Tail Suspension Test

The tail of each mouse was secured with tape to suspend it around 30 cm above the ground. The mice were suspended for six minutes, and the computer recorded their resting time. Resting represented depression-like behavior [7].

### 4.6. Open Field Test

To assess the exploratory and anxiety-like behaviors of the mice, an open-field test was conducted in a quiet, dimly illuminated chamber with a 40 × 40 × 40 cm^3^ square arena. During each testing session, each mouse was given 6 min to freely explore the testing area. Total distance traveled, center track length, and the duration spent in the center were noted [7].

### 4.7. Tissue Preparation

At the age of 20 weeks and following behavioral tests, 1% pentobarbital (50 mg/kg) was injected intraperitoneally for anesthesia, and their blood was removed transcardially using ice-cold phosphate-buffered saline (PBS). The brains were promptly frozen for ELISA, western blotting, and RT-PCR analyses. For Nissl staining and TEM, mice were injected with 4% paraformaldehyde after perfusion with PBS, and the brains were removed and placed in the same fixative solution.

### 4.8. Western Blotting

The 20th-week brain tissues were collected, lysed in 1 mL radioimmunoprecipitation assay lysis buffer combined with 1% phosphatase inhibitor and 1% phenylmethanesulfonyl fluoride to extract proteins, and then a bicinchoninic acid method protein assay kit (BCA Kit, Thermo Fisher, Waltham, MA, USA) was used to evaluate the protein constitution. Using sodium dodecyl sulfate-polyacrylamide gel electrophoresis at a concentration range of 7.5% to 15%, forty micrograms of protein were separated. The protein was subsequently deposited onto polyvinylidene fluoride (PVDF) membranes. The tissues were submerged for one hour at room temperature in 5% skim milk diluted in Tris-buffered saline with tween-20 (TBST) (50 mM Tris/HCL, pH 7.6, 0.1% Tween-20, and 150 mM NaCl) and incubated 4 °C overnight with the indicated primary antibodies: anti-GPX4 (1:1000, Abcam, 125066, Cambridge, UK), anti-ACSL4 (1:1000, Abcam, 155282), anti-COX2 (1:1000, Abcam, 62331), and anti-β-actin (1:5000, Abcam, 8227). The PVDF membranes were rinsed with TBST three times, each for five minutes, followed by an incubation at room temperature for one hour with horseradish peroxidase-conjugated secondary antibodies. The immunoreactive blots were examined utilizing the ECL Plus chemiluminescence reagent kit (Amersham Bioscience, Arlington Heights, IL, USA), and ImageJ Version 1.53a was used to quantify the gray values.

### 4.9. TEM

The mice were sacrificed at the 20th week and then injected transcardially with 4% paraformaldehyde and normal saline. Briefly, 1 mm^3^ slices of hippocampus were submerged in 2.5% glutaraldehyde for 24 h at 4 °C before being postfixed for an hour in 1% osmium tetroxide. The samples were then stained with 2% uranyl acetate and subsequently dehydrated in a series of ethanols for 10 min each. After being embedded overnight in acetone at a concentration of one hundred percent, the samples were sliced to a thickness of 100 nanometers and stained with uranyl acetate and lead citrate at concentrations of two percent. Using a Philips Tecnai 10 TEM, images were collected.

### 4.10. GPX Activity, LPO, GSH, MDA, and SOD Measurement

Fresh brain tissue samples were gathered and subsequently processed into a homogenate at a concentration of 1–10% with precooled PBS. According to the manufacturer’s instructions, the Glutathione Peroxidase Assay Kit (Abcam, 102530) was used to analyze the activity of GPX. The LPO level was analyzed using the Lipid Peroxidation Assay Kit (Nanjing Jiancheng Bioengineering Institute, A106, Nanjing, China), the glutathione (GSH) level was analyzed using the GSH and GSSG Assay Kit (Beyotime, S0053), the MDA level was analyzed using the Lipid Peroxidation Malondialdehyde Assay Kit (Beyotime, S0131S, Shanghai, China), and the SOD level was analyzed utilizing the Superoxide Dismutase Assay Kit (Nanjing Jiancheng Bioengineering Institute, A001).

### 4.11. ELISA

Based on the instructions of the manufacturer, mouse IL-1β, TNF-α, and IL-6 ELISA kits (Boster Biological Technology, Wuhan, China) were used to detect differences in inflammatory cytokines after treatment. Using a competitive ELISA kit (Cell Biolabs, San Diego, CA, USA), the concentration of 4-hydroxynonenal (4-HNE), a biomarker of lipid peroxidation, was measured.

### 4.12. Nissl Staining

We conducted Nissl staining to detect neuronal survival. The slices were rinsed and then incubated at room temperature for 30 min in a 0.5% cresol violet (Sigma-Aldrich, Darmstadt, Germany) solution. The sections were sequentially dehydrated in ethanol and cleaned in xylene. The slices of brain tissue were fixed using Permount and cover-slipped. Compared with normal neurons, the damaged neurons shrank or contained vacuoles. Under a light microscope, fields in the hippocampus and the temporal cortex were viewed, and the quantity of surviving neurons was recorded by a researcher who was blinded to the treatment group.

### 4.13. RT-PCR

Total mRNA was isolated from tissues utilizing a TRIzol™ Plus RNA Purification Kit according to the manufacturer’s instructions, and the amount of extracted RNA was measured utilizing ultraviolet absorbance at 260 nm. Each sample’s total RNA was then used to synthesize cDNA at 37 °C for 15 min, followed by 85 °C for 5 s using the PrimeScript RT Master Kit (Takara, RR420A, Kusatsu, Japan) per the manufacturer’s instructions.

RT-PCR was performed using a SYBR Premix Ex Taq™ Kit (Takara, RR036A) in an Applied Biosystems 7500 RT-PCR System (Life Technologies, California, CA, USA) as follows: 95 °C for 20 s, and 40 amplification cycles at 95 °C for 3 s and 60 °C for 30 s. The 2^−∆∆CT^ approach was utilized to determine the relative mRNA level of each target gene normalized to β-actin.

### 4.14. Statistical Analysis

SPSS software (Version 23.0) and GraphPad Prism (Version 8.0) were used for statistical analysis. The data were summarized using the following formula: mean ± standard deviation (SD). A *t*-test was employed to compare two groups in which the data distribution was normal. We employed ANOVA and Tukey’s post hoc test in order to compare the statistical differences among groups. To compare two groups, the Mann–Whitney U test was utilized. For post hoc comparisons, the Kruskal–Wallis test and the Dunn–Bonferroni test were used to evaluate statistical comparisons between various groups. The significance level was set at *p* < 0.05.

## 5. Conclusions

Our study found that ferroptosis plays a crucial role in the pathogenesis of NPSLE. Moreover, ACSL4 is considered an essential target of ferroptosis, and its inhibition enhances recovery from behavioral deficits in NPSLE via regulating ferroptosis.

## Data Availability

The raw data supporting the conclusions of this article will be made available by the authors without undue reservation.
